# Prevalence and correlates of Kaposi’s sarcoma-associated herpesvirus and herpes simplex virus type 2 infections among adults: evidence from the NHANES III data

**DOI:** 10.1186/s12985-021-01731-9

**Published:** 2022-01-06

**Authors:** Xin Zhang, Yiyun Xu, Yi Li, Huangbo Yuan, Zhenqiu Liu, Tiejun Zhang

**Affiliations:** 1 Department of Epidemiology, School of Public Health, Fudan University, Shanghai, 200032 China; 2 Key Laboratory of Public Health Safety (Fudan University), Ministry of Education, Shanghai, China; 3 State Key Laboratory of Genetic Engineering and Collaborative Innovation Center for Genetics and Development, School of Life Sciences, Fudan University, Shanghai, 200438 China; 4 Human Phenome Institute, Fudan University, Shanghai, China; 5 Yiwu Research Institue, Fudan University, Yiwu, China

**Keywords:** KSHV, HSV-2, Risk factor, NHANES

## Abstract

**Background:**

Kaposi’s sarcoma-associated herpes virus (KSHV) prevalence and risk factors exhibit considerable variations across populations in different geographic regions. Determinants and the transmission routes of KSHV infection are uncertain. We seek to identify the possible risk factors and the transmission routes of KSHV infection in non-endemic areas.

**Methods:**

We collected annual cases and seroprevalence of KSHV and herpes simplex virus type 2 (HSV-2) from the NHANES III sampled individuals from the US general population (1988–1994). We included 13,179 and 10,720 individuals with available remaining serum samples of KSHV and HSV-2. Logistic regression was employed to explore potential risk factors for the seropositivity.

**Results:**

The seroprevalence was 2.05% for KSHV infection and 31.03% for HSV2 infection among this population. All risk factors of sexual behaviors included were strongly associated with HSV-2 positive, however, only MSM had an approximately fivefold increased risk of KSHV infection (OR = 4.71; 95%CI 1.61 11.30). Mexican Americans (2.51%) and older (chi-square_trend_ =  − 6.71, *P* < 0.001) individuals had a higher risk of KSHV infection. After adjustment, individuals with higher level of education and economic status had lower KSHV infection.

**Conclusions:**

In non-endemic areas, KSHV transmission may be related to sexual activity in men, especially in male homosexuals. Higher education level and economic status are protective factors for KSHV infection.

## Introduction

Kaposi’s sarcoma-associated herpes virus (KSHV) also referred as human herpesvirus-8 (HHV-8), is the etiologic agent for the occurrence of Kaposi's sarcoma (KS), Primary effusion lymphoma (PEL) and Multicentric Castleman's disease (MCD) [1–3]. Since its initial discovery [4], the molecular biology, epidemiology and pathological characteristics of this virus have been extensively studied. KSHV infection is not ubiquitous with seroprevalence, risk factors and transmission routes exhibiting considerable variations across populations in different geographic regions [5, 6]. KSHV spreads via saliva contacting during childhood in endemic areas, while KSHV transmission could be associated with homosexual contact in non-endemic countries [6–8]. However, there are conflicting findings about specific sexual behaviors that might be responsible for the transmission of the virus and no consensus about modes of acquisition [7]. In North America and Europe, these non-endemic areas, KSHV infection is commonly found among HIV positive individuals, especially in the men who have sex with men (MSM) [9], and the relationship with the number of recent male sex partners is consistent with transmission through intimate contact between men [10].

Herpes simplex virus type 2 (HSV-2), the most common sexually transmitted agent [11], is also an opportunistic infection in HIV/AIDS patients. Both KSHV and HSV-2 are herpesviruses establishing life-long infections in humans [12], and acute infection, frequent reactivations from a previous infection or symptomatic episodes in the immunosuppressed individuals can be devastating. The fact that the two herpesviruses are common infectious agents among MSM [13], suggesting they may share similar transmission routes. Previous researches also had shown that demographic variables (age, gender, race), behavioral risk factors (sexual behavior, intravenous drug use), and a history of diseases may affect the risk of KSHV infection and associated disease progression [14, 15], but conclusions were inconclusive. Therefore, further data to compare the epidemiologic feature of these two herpesviruses in a same group would be valuable.

In the present study, we used data from the National Health and Nutrition Examination Survey III (NHANES III), a large, population-based, cross-sectional study to explore the epidemiology of KSHV and HSV-2 in the US general adult population. Through comparing the epidemiologic characteristics of these two viruses, we aimed to advance the understanding of the potential risk factors and transmission routes of KSHV, especially sexual transmission, using a nationally representative sample.

## Materials and methods

### Data collection

The characteristics and seroprevalence of KSHV and HSV-2 were collected from the NHANES III (https://wwwn.cdc.gov/nchs/nhanes/nhanes3/Default.aspx), which was a complex, multistage probability designed cross-sectional survey conducted during 1988–1994 by the National Center for Health Statistics, US Centers for Disease Control and Prevention. Data were obtained through household interviews, standardized physical examinations, and collection of biological samples at special mobile examination centers. These surveys included 39,695 individuals randomly sampled through a complex, multistage probability design [16].

Of all NHANES III participants, 33,994 were interviewed. We included a total of 18,080 individuals aged 18 to 80 years. Individuals without examination were excluded (n = 1579). KSHV antibody was tested on 13,179 participants with available remaining serum samples, and HSV-2 antibody was tested of stored sera specimens from 10,720 participants.

### Laboratory methods

The serum samples were stored at the NHANES surplus biorepository at − 80 °C. KSHV antibody testing was performed using ELISA to detect antibodies to the KSHV K8.1 structural glycoprotein (expressed during lytic viral replication) and open reading frame (ORF) 73 (also known as latency associated nuclear antigen, a viral regulatory protein expressed during latent infection). ELISA methods have been detailed elsewhere [17]. Both K8.1 and ORF73 were tested because some infected individuals, including those with KS, make antibodies only to lytic or latent antigens [18]. Sera were tested for HSV-2 antibodies using purified glycoproteins specific for gG-2 as antigens to detect type-specific antibodies with solid-phase enzymatic immunodot assays [19]. The sensitivity of the immunodot test for recurrent, culture-proved genital HSV-2 infection is over 98%, and the specificity is over 99% [20].

### Statistical analysis

KSHV and HSV-2 seroprevalence were examined in specific subgroups of NHANES III subjects. Subjects with missing data were excluded. The participants were chosen to be 18–80 years old due to the large bias of Mexican Americans over 80 years old. Race/ethnicity was defined as non-Hispanic white, non-Hispanic black, Mexican American and others. Persons not fitting in the previous three categories were included in the others. Household size represents the total number of persons living in a single dwelling unit, both related and unrelated. Self-reported history of genital herpes was also evaluated through interview data. And participants were asked “Has a doctor or other healthcare professional ever told you that you had genital herpes?” The sexual history was asked among all participants 17–59 years old through a face-to-face interview. The seroprevalence according to whether or not have sexual behaviors, the time of first sexual intercourse, lifetime and the past year number of sex partners, and MSM activity was examined. MSM activity was presented if a man reported at least 1 male sex partner [17].

Descriptive statistical analysis and measures of association were performed using the R programme (version 3.5.1, R core team, Vienna, Austria). Categorical variables were described as numbers or proportions. Differences for ordinal categorical variables were tested using Cochran Armitage tests. Odds ratios (OR) and 95% confidence intervals (CI) for all potential risk factors of KSHV and HSV-2 seropositivity among the subjects were calculated by univariate logistic regression models. Then we acquired adjusted odds ratios (aORs) after adjustment for age groups, sex, and race/ ethnicity. A two-sided p-value of < 0.05 was used as a measure of significance of the associations.

## Results

The 16,501 participants were predominately middle age (61.56% under 50 years), non-Hispanic whites (38.29%) and female (53.25%). Majority (69.09%) of them had received middle school education and with median income (44.08%). Most (60.42%) were married or living as married and 59.12% were with employment.

### Characteristics and their associations with KSHV and HSV-2 infection

**Fig. 1:**
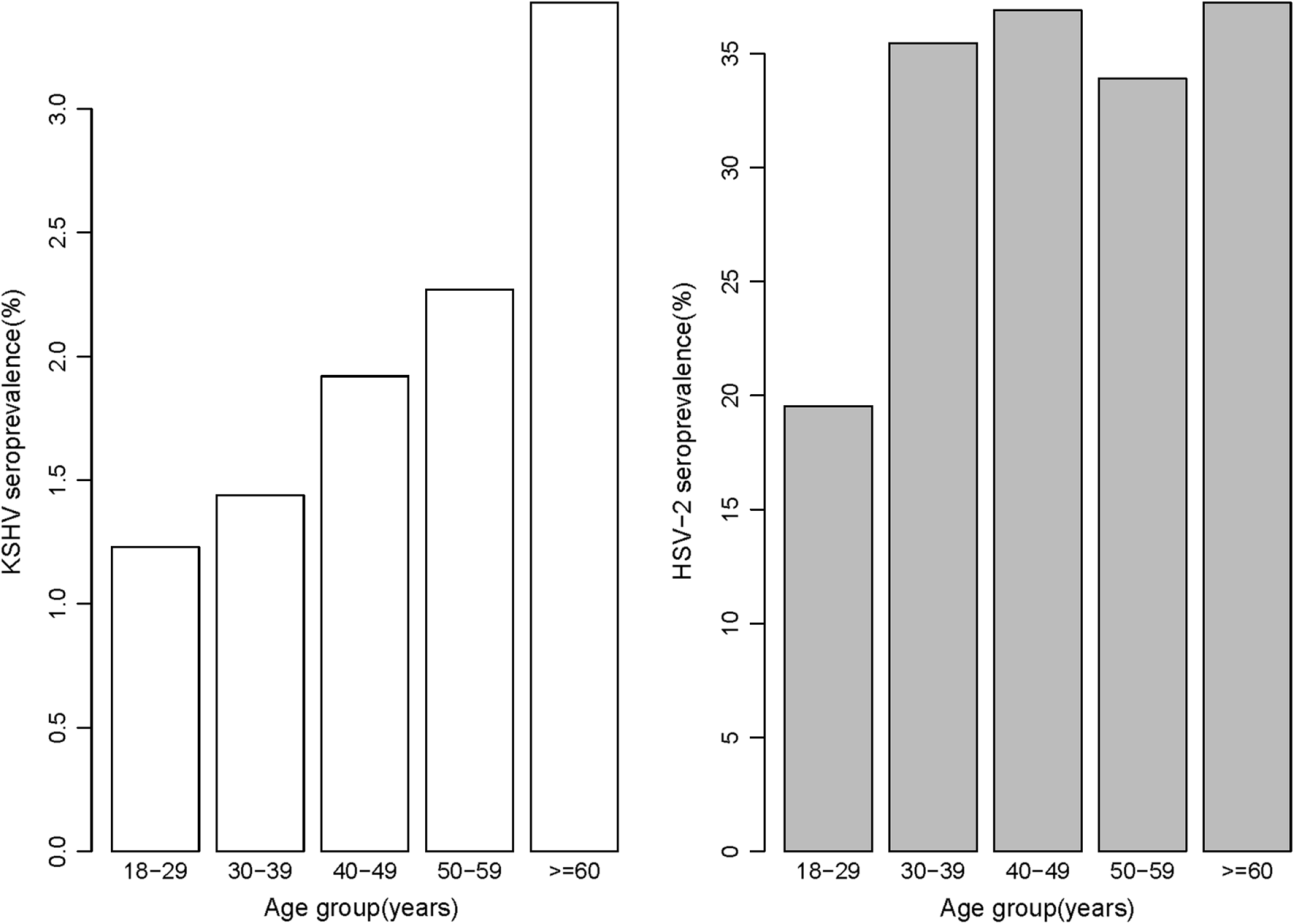
The age distribution of KSHV and HSV2 prevalence among participants

Of the 13,179 participants with available KSHV remaining serum samples, 270 individuals were K8.1 seropositive, yielding a seroprevalence estimate of 2.05%. When detecting the antibodies to the ORF73, 211 (1.60%) were KSHV seropositive. As shown in Table 1, a significant difference for K8.1 seroprevalence was detected across age group (chi-square_trend_ = -6.71, *P* < 0.001). Infection rates increased from 1.23% among 18–29 years old to 3.43% among 60–80 years old (Fig. 1). K8.1 seroprevalence was higher among Mexican Americans (2.51%) and non-Hispanic blacks (2.14%) than among non-Hispanic whites (1.67%). Individuals with higher education level (chi-square_trend_ = 4.35, *P* < 0.001) and economic status (chi-square_trend_ = 2.97, *P* = 0.001) had lower K8.1 seroprevalence. Compared to non-smoking participants, smokers had a higher infection rate, regardless of whether they had quitted. We observed no statistically significant associations with occupation and marital status after adjusting the age, sex and race. Patterns for ORF73 seroprevalence were similar to those for K8.1 seroprevalence, but differences across subgroups were less distinct and did not reach statistical significance (data not shown).

**Table1:** Socio-demographic and their associations with KSHV infection and HSV-2 infection in study participants

Characteristic	All subjects, No (%)	KSHV			HSV-2		
		Infection, n/N (%)	OR (95% CI)	aORs^$^ (95% CI)	Infection, n/N (%)	OR (95% CI)	aORs^$^ (95% CI)
All subjects	16,501	2.05			31.03		
Gender							
Male	7714(46.75)	2.13	1.00	1.00	24.90	1.00	1.00
Female	8787(53.25)	1.98	0.93 (0.73,1.18)	0.95 (0.75,1.21)	36.96	1.77* (1.63,1.92)	1.84* (1.68,2.01)
*P*			0.555	0.613		< 0.001*	< 0.001*
Age							
18–29 years	4238(25.68)	1.23	1.00	1.00	19.53	1.00	1.00
30–39 years	3333(20.20)	1.44	1.18 (0.76,1.82)	1.23 (0.79,1.90)	35.45	2.26* (2.01,2.55)	2.48* (2.19,2.81)
40–49 years	2588(15.68)	1.92	1.58* (1.02,2.43)	1.67* (1.08,2.57)	36.90	2.41* (2.13,2.73)	2.92* (2.56,3.33)
50–59 years	1869(11.33)	2.27	1.87* (1.17,2.95)	2.16* (1.35,3.43)	33.90	2.11* (1.79,2.49)	3.07* (2.57,3.66)
60–80 years	4473(27.11)	3.43	2.86* (2.02,4.14)	3.37* (2.36,4.90)	37.24	2.45* (2.16,2.77)	3.81* (3.32,4.38)
*P*			< 0.001*	< 0.001*		< 0.001*	< 0.001*
Race/ethnicity							
Non-Hispanic white	6318(38.29)	1.67	1.00	1.00	18.84	1.00	1.00
Non-Hispanic black	4840(29.33)	2.14	1.29 (0.95,1.75)	1.59* (1.17,2.17)	50.09	4.32* (3.89,4.81)	5.52* (4.92,6.19)
Mexican–American	4664(28.26)	2.51	1.52* (1.13,2.05)	1.96* (1.44,2.66)	26.84	1.58* (1.41,1.77)	2.00* (1.78,2.25)
Other	679(4.11)	2.00	1.20 (0.62,2.13)	1.42 (0.73,2.52)	29.11	1.77* (1.40,2.23)	2.16* (1.69,2.74)
*P*			0.017*	< 0.001*		< 0.001*	< 0.001*
Household size							
1–4	12,230(74.12)	2.10	1.00	1.00	31.18	1.00	1.00
5–9	3875(23.48)	1.88	0.90 (0.66,1.19)	0.99 (0.72,1.35)	30.69	0.98 (0.89,1.07)	0.96 (0.86,1.06)
≥ 10	396(2.40)	2.24	1.07 (0.45,2.13)	1.15 (0.48,2.34)	30.14	0.95 (0.73,1.23)	1.03 (0.77,1.36)
*P*			0.619	0.741		0.562	0.771
Education							
0 years	424(2.59)	6.80	1.00	1.00	39.46	1.00	1.00
1–12 years	11,328(69.09)	2.11	0.30* (0.19,0.49)	0.46* (0.28,0.79)	33.03	0.76 (0.58,1.00)	0.84 (0.63,1.13)
≥ 13 years	4645(28.33)	1.50	0.21* (0.13,0.36)	0.39* (0.22,0.70)	25.73	0.53* (0.40,0.71)	0.64* (0.47,0.87)
*P*			< 0.001*	0.005*		< 0.001*	< 0.001*
Poverty income ratio							
≤ 1.3	4987(33.36)	2.57	1.00	1.00	37.36	1.00	1.00
1.3—3.5	6589(44.08)	1.85	0.71* (0.54,0.95)	0.73* (0.55,0.98)	29.91	0.72* (0.65,0.79)	0.79* (0.71,0.88)
> 3.5	3371(22.55)	1.57	0.60* (0.42,0.86)	0.65* (0.43,0.95)	23.78	0.52* (0.46,0.59)	0.65* (0.57,0.75)
*P*			0.003*	0.005*		< 0.001*	< 0.001*
Occupation							
Unemployment	6744(40.88)	2.59	1.00	1.00	34.81	1.00	1.00
Employment	9754(59.12)	1.70	0.65* (0.51,0.83)	0.92 (0.69,1.22)	28.97	0.76* (0.70,0.83)	0.97 (0.87,1.07)
*P*			< 0.001*	0.358		< 0.001*	0.278
Marital status							
Never married	3337(20.26)	1.76	1.00	1.00	31.27	1.00	1.00
Married/living as married	9950(60.42)	1.87	1.07 (0.77,1.50)	0.69 (0.47,1.02)	31.20	1.00 (0.90,1.11)	0.96 (0.86,1.08)
Separated/divorced/widowed	3182(19.32)	2.94	1.70* (1.18,2.47)	0.93 (0.61,1.45)	30.32	0.96 (0.84,1.08)	0.93 (0.81,1.06)
*P*			0.003*	0.781		0.478	0.346
Smoking status							
No-smoking	8209(49.75)	1.73	1.00	1.00	27.85	1.00	1.00
On-smoking	4438(26.90)	1.95	1.13 (0.83,1.52)	1.21 (0.88,1.65)	36.12	1.47* (1.33,1.61)	1.71* (1.53,1.90)
Quit-smoking	3853(23.35)	2.86	1.67* (1.26,2.21)	1.44* (1.06,1.95)	31.68	1.20* (1.08,1.34)	1.41* (1.24,1.59)
*P*			< 0.001*	0.023*		< 0.001*	< 0.001*
Drinking status							
No-drinking	2838(17.98)	2.33	1.00	1.00	29.03	1.00	1.00
Drinking	12,943(82.02)	2.03	0.87 (0.65,1.19)	1.07 (0.77,1.47)	31.26	1.11 (1.00,1.24)	1.54* (1.36,1.75)
*P*			0.369	0.602		0.062	< 0.001*

*KSHV* Kaposi’s sarcoma-associated herpesvirus, the KSHV seroprevalence was used antibodies to K8.1; *OR* Odds ratio; *aORs* adjusted odds ratios; *CI* confidence interval

^*^Statistically significant association

^$^OR and 95%CI adjusted for age, sex and race/ethnicity

Overall, the seroprevalence of HSV-2 was 31.03% (3326 out of 10,720). Of those testing positive for HSV-2 infection, 95.12% reported never been diagnosed with genital herpes. Female (36.96%) and non-Hispanic blacks (50.09%) had a higher risk of HSV-2 infection. There was a strong association between HSV-2 seropositivity and age, with prevalence generally rising rapidly between the ages of 18 and 39 years before becoming stable. Compared with KSHV infection, drinking was also correlated with HSV-2 seropositivity, in addition to education, economic status, and smoking status. Drinking increased the risk of HSV-2 infection after adjustment (OR = 1.54; 95%CI 1.36 1.75).

### Co-infection and their associations with KSHV and HSV-2 infection

The overall co-infection rates of Hepatitis B virus (HBV, HBsAg), Hepatitis C virus (HCV), Herpes simplex virus type 1 (HSV-1), HSV-2 and Cytomegalovirus (CMV) were 7.94%, 2.21%, 1.99%, 2.41% and 2.40%, respectively. Univariate logistic regression analyses indicated that both HSV-2 and CMV infection were significantly associated with K8.1 seropositivity. Those who were infected with HSV-2 (OR 1.58, 95% CI 1.09 2.27) or CMV (OR 2.12, 95% CI 1.37 3.43) were more likely to be infected with KSHV. Such associations were still significant after adjusting sex, age, race, education level and economic status for potential confounders (Table 2). However, there was no statistically significant association between KSHV infection and HBV, HCV and HSV-1 infection. Different with KSHV co-infection, participants infected with HCV were more likely to be infected with HSV-2 (OR 2.16, 95% CI 1.63 2.85).

**Table 2:** Serostatus of HBsAg, HCV, HSV-1 and CMV and Co-infection by KSHV and HSV-2 in study participants

Characteristic	KSHV			HSV-2		
	Infection, n/N (%)	aORs^$^ (95% CI)	*P*	Infection, n/N (%)	aORs^$^ (95% CI)	*P*
All subjects	2.05			31.03		
HBV						
No	3.69	1.00	0.137	56.06	1.00	0.618
Yes	7.94	2.45 (0.65,7.27)		51.85	1.18 (0.62,2.28)	
HCV						
No	2.04	1.00	0.612	30.48	1.00	< 0.001*
Yes	2.21	1.22 (0.51,2.45)		50.37	2.16 (1.63,2.85)	
HSV-1						
No	1.20	1.00	0.194	27.17	1.00	0.159
Yes	1.99	1.37 (0.87,2.26)		32.13	0.92 (0.81,1.03)	
HSV-2						
No	1.54	1.00	0.014*	–	–	–
Yes	2.41	1.58 (1.09,2.27)		–	–	–
KSHV						
No	–	–	–	30.65	1.00	0.006*
Yes	–	–	–	41.36	1.66 (1.15,2.37)	
CMV						
No	0.88	1.00	0.001*	17.43	1.00	< 0.001*
Yes	2.40	2.12 (1.37,3.43)		35.55	1.59 (1.40,1.81)	

*KSHV* Kaposi’s sarcoma-associated herpesvirus, the KSHV seroprevalence was used antibodies to K8.1; *HSV-2* Herpes simplex virus type 2; *HBV* Hepatitis B virus; *HCV* Hepatitis C virus; *HSV-1* Herpes simplex virus type 1; *CMV* Cytomegalovirus; *aORs* adjusted odds ratios; *CI* confidence interval

^$^OR and 95%CI adjusted for age, sex, race/ethnicity, education level and economic status as shown in Table 1

Seroprevalence of HBsAg, HCV, HSV-1, HSV-2, KSHV and CMV, respectively

^*^Statistically significant association

### Sexual behaviors and their associations with KSHV and HSV-2 infection

All sexual behaviors included were strongly associated with HSV-2 infection, confirmed HSV-2 was a common sexually transmitted infection (Table 3). In contrast to HSV-2, the prevalence of KSHV did not follow the pattern expected of a sexually transmitted infection. After adjusting the age, the duration of sexual activity did not show a significant association with KSHV seropositivity. Only participants with homosexual activity showed an elevated risk of KSHV infection. Of all subjects, 83 reported a history of homosexual activity (weighted prevalence, 1.07% among all male participants), and 5 out of 83 were K8.1 positive. Participants with MSM activity increased the risk of KSHV infection fourfold (OR=4.98; 95%CI 1.69 11.77; P<0.001).

**Table 3:** Sexual behaviors and their associations with KSHV infection and HSV-2 infection in study participants

Characteristic	All subjects, No (%)	KSHV			HSV-2		
		Infection, n/N (%)	OR (95% CI)	aORs^$^ (95% CI)	Infection, n/N (%)	OR (95% CI)	aORsF^$^ (95% CI)
Sexual							
No sex	344(2.98)	1.82	1.00	1.00	5.93	1.00	1.00
Sex	11,185(97.20)	1.62	0.89 (0.40,2.52)	0.72 (0.31,2.06)	30.27	6.89* (4.23,12.15)	4.64* (2.82,8.26)
*P*			0.796	0.532		< 0.001*	< 0.001*
Initial sexual age							
≤ 18 years	8339(74.56)	1.74	1.00	1.00	32.39	1.00	1.00
19–29	2776(24.82)	1.28	0.73 (0.48,1.08)	0.72 (0.47,1.08)	23.71	0.65* (0.58,0.73)	0.56* (0.49,0.64)
≥ 30	70(0.63)	1.72	0.99 (0.06,4.56)	0.81 (0.05,3.85)	25.00	0.70 (0.34,1.37)	0.47* (0.22,0.93)
* P*			0.154	0.083		< 0.001*	< 0.001*
Sex partners							
1	2418(21.91)	1.40	1.00	1.00	16.40	1.00	1.00
2–4	3481(31.54)	1.61	1.15 (0.72,1.86)	1.13 (0.70,1.84)	30.26	2.21* (1.90,2.58)	2.38* (2.02,2.80)
5–9	2249(20.38)	1.81	1.29 (0.78,2.15)	1.23 (0.72,2.09)	35.60	2.82* (2.40,3.32)	3.48* (2.91,4.18)
≥ 10	2890(26.18)	1.66	1.18 (0.73,1.95)	1.06 (0.61,1.84)	35.90	2.85* (2.45,3.34)	4.85* (4.03,5.85)
*P*			0.463	0.574		< 0.001*	< 0.001*
The duration of sexual activity							
0–20	6243(54.15)	1.25	1.00	1.00	24.14	1.00	1.00
21–30	2946(25.55)	2.07	1.67* (1.15,2.40)	1.53 (0.90,2.58)	36.91	1.84* (1.65,2.05)	1.47* (1.25,1.72)
31–40	1812(15.72)	1.95	1.57* (1.00,2.42)	1.29 (0.58,2.81)	35.90	1.76* (1.53,2.03)	1.83* (1.43, 2.34)
≥ 40	528(4.58)	2.57	2.08* (1.00,3.89)	1.58 (0.54,4.36)	40.53	2.14* (1.66,2.76)	2.46* (1.69,3.59)
*P*			0.004*	0.320		< 0.001*	0.002*
Sex partners in the past year							
0	1110(9.92)	2.62	1.00	1.00	32.30	1.00	1.00
1	8188(73.20)	1.52	0.57* (0.37,0.92)	0.62* (0.40,1.01)	28.83	0.85 (0.72,1.00)	1.01 (0.84,1.21)
2–4	1588(14.20)	1.37	0.52* (0.28,0.95)	0.54 (0.28,1.01)	35.17	1.14 (0.94,1.38)	1.55* (1.25,1.94)
5–9	219(1.96)	2.84	1.09 (0.36,2.67)	1.08 (0.35,2.77)	33.33	1.05 (0.73,1.49)	1.78* (1.20,2.60)
≥ 10	81(0.72)	–	–	–	41.27	1.47 (0.86,2.48)	2.07* (1.17,3.64)
*P*			0.132	0.205		0.002*	< 0.001*
Sexual preference							
No-MSM	4922(98.34)	1.63	1.00	1.00	22.30	1.00	1.00
MSM	83(1.66)	7.25	4.71* (1.61,11.03)	4.98* (1.69,11.77)	39.68	2.29* (1.36,3.80)	2.85* (1.65,4.87)
*P*			0.001*	< 0.001*		0.001*	< 0.001*

*KSHV* Kaposi’s sarcoma-associated herpesvirus, the KSHV seroprevalence was used antibodies to K8.1; *OR* Odds ratio; *aORs* adjusted odds ratios; *CI* confidence interval

^*^Statistically significant association

^$^OR and 95%CI adjusted for age, sex and race/ethnicity as shown in Table 1

## Discussion

Despite consistent increasing understanding of KSHV biology and its clinical manifestations, progress has not been significantly made in its epidemiology which in turn hampered the management of KSHV-associated diseases and public health. In this study, we based on a large general population to explore the epidemiologic features of KSHV infection. The seropositivity of KSHV was 2.05%, higher than the previous study [17]. And the results suggested that KSHV appears to be sexually transmitted in MSM, this high risk population.

We explored the potential correlates of sexual behaviors with KSHV and HSV-2 infection and found that KSHV transmission may be related to sexual activity in men, especially in male homosexuals. Our results showed MSM had an approximately fivefold increased risk of KSHV infection (OR = 4.98), which was generally consistent with previous studies [8, 21, 22]. Whereas, we did not find evidence that heterosexual transmission of KSHV infection. Compared with heterosexuals, homosexual individuals were more likely to have oral sex and multiple sexual partners [23]. While KSHV can be detected commonly in the saliva of infected subjects, indicating that saliva is the most likely source of KSHV during transmission between MSM [24, 25]. Our results did not show a clear between the number of sexual partners and KSHV seropositivity (P = 0.574). This may be affected by the methods of data acquisition. The data on sexual behaviors were obtained through interviews with the general population, which may cause some selection biases.

As expected, there was an increased HSV-2 prevalence with earlier initial sexual age and number of sexual partners in life. Meanwhile, HSV-2 infection risk increased about threefold (OR = 2.85) among MSM. High prevalence of the two viruses among MSM suggests that the two viruses may facilitate the acquisition of each other through sexual contacts with this population and further supported KSHV transmission may be related to male homosexuals. HSV-2 infection can contribute to the sexual transmission of KSHV and may also be associated with reactivation of KSHV latent infection. Previous studies in Northern Cameroon showed similar results [7]. HSV-2 infection is known to recruit at the site of replication white blood cells including B and CD8 lymphocyte that can be infected by KSHV. In addition, reactivation of HSV-2 might increase the load at mucosal and systemic levels of other viruses including KSHV, HIV [26], and HCV [27]. Viral shedding and transmission to sex partners can occur in the absence of symptoms or a noticeable lesion [28]. Noticeably, our study revealed that 95.12% of HSV-2 infections were asymptomatic or unrecognized, and this could exacerbate the transmission of KSHV. We also found that HSV-2 positive subjects were more likely to be infected with KSHV (OR = 1.58), this association implies that HSV-2 and KSHV may share similar transmission routes.

Furthermore, we have also confirmed KSHV infection was associated with increasing age, compared with subjects between 18–29 years old, the risk among 50–59 years old was increased about twofold (OR = 2.27) and among 60–80 years old was increased about threefold (OR = 3.43). Unlike the relatively stable age pattern of HSV-2 seroprevalence, the prevalence of KSHV increases steadily with age, suggesting that KSHV may not mainly through sexually transmitted. The role of education level and economic status against KSHV have been explored by other investigators, however, the results are controversial [29]. Our study suggests that higher education level and higher economic status are protective factors for KSHV infection. Participants with higher education and economic level may pay more attention to self-protection [30]. Regarding the household size, we did not find the correlation with KSHV seroprevalence, however, we did not know whether people living together share public utensils. Thus, we cannot infer the possible transmission route through saliva from the household size.

Defining modes of transmission of KSHV remains a challenge, especially in non-endemic areas. In this study, we were able to compare the well-known pattern of sexually related risk factors for HSV-2, with that seen for KSHV in a community at risk of both. The implication of our findings is that KSHV appears to be sexually transmitted in MSM, although transmission among heterosexuals is less likely. Generally, oral transmission is the main route of KSHV infection in both endemic and non-endemic areas. The KSHV infection occurs primarily in children in endemic areas, but tends to afflict adults in non-endemic areas, especially in homosexual male. Prevention priority should be given to children in endemic areas, while attention should be paid in preventing KSHV infection among MSM group in non-endemic areas.

Some limitations should be noted here. First, there is a lack of standard KSHV serological assay. We are based on the results from the EIAs, which may be different with other methods. Second, NHANES III lacks data on specific sexual practices that are unavailable, which prevented us from exploring potential routes of sexual transmission in more details.

## Conclusion

In non-endemic areas, KSHV transmission may be related to sexual activity in men, especially in male homosexuals. Our results clarify the transmission routes of KSHV infection, which would ultimately help the development of interventions that prevent infection.

## Data Availability

The datasets analysed during the current study were collected from the NHANES III (https://wwwn.cdc.gov/nchs/nhanes/nhanes3/Default.aspx), which was a complex, multistage probability designed cross-sectional survey conducted during 1988–1994 by the National Center for Health Statistics, US Centers for Disease Control and Prevention.

## Abbreviations

KSHV: Kaposi’s sarcoma-associated herpes virus
HHV-8: Human herpesvirus-8
KS: Kaposi's sarcoma
PEL: Primary effusion lymphoma
MCD: Multicentric Castleman's disease
MSM: Men who have sex with men
HSV-2: Herpes simplex virus type 2
NHANES III: National Health and Nutrition Examination Survey III
ORF: Open reading frame
